# Identification and validation of hub genes of synovial tissue for patients with osteoarthritis and rheumatoid arthritis

**DOI:** 10.1186/s41065-021-00201-0

**Published:** 2021-09-28

**Authors:** Yanzhi Ge, Zuxiang Chen, Yanbin Fu, Xiujuan Xiao, Haipeng Xu, Letian Shan, Peijian Tong, Li Zhou

**Affiliations:** 1 The First Affiliated Hospital, Zhejiang Chinese Medical University, Hangzhou, Zhejiang P. R. China; 2 Guangdong Provincial People’s Hospital, Guangdong Academy of Medical Sciences, School of Medicine, South China University of Technology, Guangzhou, Guangdong P. R. China; 3 College of Pharmacy, Zhejiang Chinese Medical University, Hangzhou, P. R. China; 4 The Third Clinical Medical College, Zhejiang Chinese Medical University, Hangzhou, Zhejiang P. R. China

**Keywords:** Osteoarthritis, Rheumatoid arthritis, Differentially expressed genes, Bioinformatics analysis, Immune infiltration

## Abstract

**Background:**

Osteoarthritis (OA) and rheumatoid arthritis (RA) were two major joint diseases with similar clinical phenotypes. This study aimed to determine the mechanistic similarities and differences between OA and RA by integrated analysis of multiple gene expression data sets.

**Methods:**

Microarray data sets of OA and RA were obtained from the Gene Expression Omnibus (GEO). By integrating multiple gene data sets, specific differentially expressed genes (DEGs) were identified. The Gene Ontology (GO) functional annotation, Kyoto Encyclopedia of Genes and Genomes (KEGG) pathways and protein–protein interaction (PPI) network analysis of DEGs were conducted to determine hub genes and pathways. The “Cell Type Identification by Estimating Relative Subsets of RNA Transcripts (CIBERSORT)” algorithm was employed to evaluate the immune infiltration cells (IICs) profiles in OA and RA. Moreover, mouse models of RA and OA were established, and selected hub genes were verified in synovial tissues with quantitative polymerase chain reaction (qPCR).

**Results:**

A total of 1116 DEGs were identified between OA and RA. GO functional enrichment analysis showed that DEGs were enriched in regulation of cell morphogenesis involved in differentiation, positive regulation of neuron differentiation, nuclear speck, RNA polymerase II transcription factor complex, protein serine/threonine kinase activity and proximal promoter sequence-specific DNA binding. KEGG pathway analysis showed that DEGs were enriched in EGFR tyrosine kinase inhibitor resistance, ubiquitin mediated proteolysis, FoxO signaling pathway and TGF-beta signaling pathway. Immune cell infiltration analysis identified 9 IICs with significantly different distributions between OA and RA samples. qPCR results showed that the expression levels of the hub genes (*RPS6*, *RPS14*, *RPS25*, *RPL11*, *RPL27*, *SNRPE, EEF2* and *RPL19*) were significantly increased in OA samples compared to their counterparts in RA samples (*P* < 0.05).

**Conclusion:**

This large-scale gene analyses provided new insights for disease-associated genes, molecular mechanisms as well as IICs profiles in OA and RA, which may offer a new direction for distinguishing diagnosis and treatment between OA and RA.

## Background

Osteoarthritis (OA) is one of the most common arthritic diseases in elders, involving multiple joints with declining joint functions. As the main cause of disability, OA has gradually increased the healthcare and societal costs in older adults [1]. Rheumatoid arthritis (RA) is one of the most common autoimmune diseases among connective tissue disorders, which typically involves small joints such as hands and feet [2]. Similar to OA, RA reduces quality of life and increases the risk of disability in affected patients, and imposes considerable financial and societal burdens on healthcare systems worldwide [3]. Both OA and RA patients have comparable symptoms, including pain, swelling, and dysfunction around the joints [4]. Current therapies for OA and RA are similar, such as oral medications with nonsteroidal anti-inflammatory drugs [5, 6], intra-articular injection with hormones and sodium hyaluronate [7, 8], and surgical intervention for end-stage lesions [9, 10]. However, the interventions are inadequate to impede the occurrence and progression of OA and RA. Although OA and RA share the similar therapeutic strategies, their pathological mechanisms are quite different. Growing evidences have demonstrated that the degeneration and loss of articular cartilage, subchondral bone remodeling, and sclerosis, and inflammation of the synovium and synovia are the main pathomorphological changes of OA [11]. In contrast, RA is characterized by an autoimmune-mediated attack in the synovial membranes of the affected joints, which leads to the deterioration of cartilage and bone [12]. To distinguish OA and RA, previous study has pointed out the importance of diagnostic accuracy [13]. Nevertheless, the specific diagnosis of OA and RA remains restricted due to their unclear pathological mechanisms.

In recent years, bioinformatics analysis of microarray data has been applied to probe the mechanisms of OA and RA, revealing some novel insights [14, 15]. Thus, integrated bioinformatics analysis of these gene expression data in multiple platforms will be conducive to discovering the potential mechanism of OA and RA. In this study, we aimed to firstly identify hub genes and related pathways involved in OA and RA, and finally screen out genes and signaling pathways to distinguish them. Functional annotations of differentially expressed genes (DEGs) and three protein–protein interaction (PPI) networks were constructed by data mining. Moreover, immune infiltration analysis was performed to investigate the differences and relationships of 22 immune cell types between OA and RA. Finally, overlapping hub genes between OA and RA were determined and verified by quantitative polymerase chain reaction (qPCR) in mouse models.

## Materials and methods

### Microarray data

The gene expression data was downloaded from the Gene Expression Omnibus (GEO) database (http://www.ncbi.nlm.nih.gov/geo). “Rheumatoid arthritis” and “Osteoarthritis” were set as the keywords to search in the GEO database, leading to a total of 8046 results in the GEO database up to December 22, 2020. The inclusion criteria of microarray data sets were as follows: (1) data sets should be messenger RNA transcriptome data; (2) samples were limited to synovial tissue.

### Integration of microarray data and screening of DEGs

Probe ID extracted from the downloaded series matrix was converted into gene symbol using Perl language (version 5.30.0). Corresponding gene symbols were merged into three groups and saved in TXT files. The quantile normalization of multiple gene expression data sets was performed with R software (version 3.6.1) to minimize the heterogeneity. Each gene was calculated by a t-test through its gene expression level and DEGs were screened employing “sva” and “limma” packages in R software. The screening thresholds were |logFC| (|log2(fold change)|) > 1 and adjust-*P* value < 0.05. Hierarchical clustering analysis of the top 100 DEGs was conducted using the “pheatmap” package in R.

### Gene ontology (GO) and pathway enrichment analysis

GO (http://geneontology.org/) enrichment analysis was employed to annotate genes. Cellular component (CC), biological processes (BP), and molecular function (MF) were generated through “enrichplot”, “DOSE” and “ggplot2” packages [16]. Kyoto Encyclopedia of Genes and Genomes (KEGG) database (https://www.kegg.jp/) was used for pathway enrichment analysis. GO and KEGG pathways of DEGs were visualized through “colorspace” and “stringi” packages. The false discovery rate < 0.05 and adjusted-*P* value < 0.05 were used as the cut-off criteria.

### Gene set enrichment analysis (GSEA)

The GSEA [17] software was downloaded from http://software.broadinstitute.org/gsea/index.jsp. To further identify the different functions of the hub genes, the GSEA analysis was conducted with merged data between OA-specific DEGs and RA-specific DEGs. The false discovery rate < 0.05 and adjusted-*P* value < 0.05 were set as the cut-off criteria.

### PPI network analysis

The Search Tool for the Retrieval of Interacting Genes (STRING) was used to evaluate and integrate PPI information of DEGs [18, 19]. Proteins included in the PPI networks were protein-encoding DEGs with a similar gene change in negative control (NC) vs. OA, NC vs. RA, and OA vs. RA. In all three groups, the minimum required interaction score with a confidence score of 0.99 was applied to build the PPI networks. Cytoscape software (version 3.5) was used to construct PPI networks of OA and RA-specific DEGs.

### Immune infiltration cells (IICs)

The Cell Type Identification by Estimating Relative Subsets of RNA Transcripts (CIBERSORT) online tool (http://cibersort.stanford.edu/) [20] was used to explore the state of OA and RA synovial membranes in preestablished 22 types of IICs. The analysis result was filtered complying with the cut-off criteria that adjusted-*P* value < 0.05, and the IICs composition of each sample was visualized using “barplot”, “corrplot” and “ggplot2” packages in R language version 3.6.1 [21].

### Animal experiments

Male C57BL/6 (Grade SPF II) mice (8 weeks old; mean body weight = 25.5 g) were provided by Shanghai Super B&K Laboratory Animal Co. Ltd. (Certificate number: SCXK (Shanghai) 2013–0016). The animal experiments were in accordance with the China legislation on the use and care of laboratory animals and approved by the Medical Norms and Ethics Committee of Zhejiang Chinese Medical University. C57BL/6 mice were randomly designated into 3 groups: OA group, RA group and NC group (*n* = 10 per group). Destabilization of the medial meniscus (DMM) was employed to establish OA model [22]. In brief, after anesthetized with 3% pentobarbital sodium (0.15 mL/100 g) intraperitoneally, the bilateral knee joints of mice were exposed through a medial capsular incision. Then, the medial meniscotibial ligament was transected, and the medial meniscus was displaced medially. Finally, the incision was washed with 20 mL saline and sutured. For the establishment of RA model, another 10 mice were randomly chosen to inject Complete Freund’s Adjuvant (CFA) at bilateral knees (10 μL per knee) [23]. Sham operation was done in parallel, with only the skin of the knee joints resected. Mice were sacrificed 8 weeks after surgery.

### Evaluation of arthritis severity and tactile sensitivity testing

To evaluate RA mice, a subjective scoring system [24] was applied in both lower limbs from the lowest grade of 0 to the highest grade of 4 (Table 1). Accordingly, the subjectivity of this score including ankylosis of the limb, arguably the severest form of arthritis, can be a concern for quantitative analysis of the RA model.

**Table 1:** Scoring system for subjective evaluation of arthritis severity

Severity	Degree of inflammation
0	No evidence of erythema and swelling
1	Erythema and mild swelling confined to the tarsals or ankle joint
2	Erythema and mild swelling extending from the ankle to the tarsals
3	Erythema and moderate swelling extending from the ankle to metatarsal joints
4	Erythema and severe swelling encompass the ankle, foot and digits, or ankylosis of the limb

In OA model group, mice were acclimated for 30 min in closed chambers before von Frey testing (UGO, USA). The surface of bilateral hind paws was stimulated with ascending force to determine tactile sensitivity. Afterwards, a rapid withdrawal of the tested paw was defined as a positive reaction, and the number of positive responses was recorded digitally [25].

### Quantitative polymerase chain reaction (qPCR) assay

The 30 synovial samples were collected from mice after being sacrificed in a CO_2_ chamber. Total RNA was extracted with TRIzol reagent and quality controlled was conducted by NanoDrop2000 spectrophotometer (Thermo Scientific, USA). Then, reverse transcription was conducted to produce cDNA. The final qPCR reaction was done in a 20 μL system, including 10 μL SYBR® Premix Ex Taq II (Tli RnaseH Plus), 0.4 μL gene specific forward primer, 0.4 μL gene specific reverse primer, 1 μL template cDNA and 8.2 μL RNase free water. The qPCR reaction was conducted with ABI QuantStudio™ 7 Flex Real-Time PCR System (Applied Biosystems, Thermo Scientific, USA), the reaction condition was as follows: 95 ºC 5 min for initial denaturation, followed by 40 cycles of denaturation at 95 ºC for 10 s, annealing and extension at 60 ºC for 30 s [26]. *β-actin* was used as an internal reference. Relative mRNA expression level was calculated with 2^−ΔΔCt^ method. Primer sequences of targeted genes were listed in Table 2.

**Table 2:** Primer sequences of target genes

Gene	Forward primer	Reverse primer
*β-actin*	5′-AGGGGCCGGACTCGTCATACT-3′	5′-GGCGGCACCACCATGTACCCT-3′
*RPS6*	5′-AGCTCCGCACCTTCTATGAGA-3′	5′-GGGAAAACCTTGCTTGTCATTC-3′
*RPS14*	5′-TGCCACATCTTTGCATCCTTC-3′	5′-ACTCATCTCGGTCAGCCTTCA-3′
*RPS25*	5′-CCCAGTAAATAAATCTGGTGGCA-3′	5′-CGGAACCTCCTTACAGAGCTT-3′
*RPL11*	5′-ATGGCGCAAGATCAAGGGG-3′	5′-GACTGTGCAGTGAACAGCAAT-3′
*RPL27*	5′-AAAGCCGTCATCGTGAAGAAC-3′	5′-GCTGTCACTTTCCGGGGATAG-3′
*RPS29*	5′-GTCTGATCCGCAAATACGGG-3′	5′-AGCCTATGTCCTTCGCGTACT-3′
*SNRPE*	5′-CAGGGCCAAAAGGTGCAGAA-3′	5′-ATTCACTTGTTCATACAGCCACA-3′
*EEF2*	5′-TGTCAGTCATCGCCCATGTG-3′	5′-CATCCTTGCGAGTGTCAGTGA-3′
*RPL10A*	5′-ATGAGCAGCAAAGTCTCACG-3′	5′-GGTCGTAGTTCTTCAGGCTGAT-3′
*RPL19*	5′-ATGAGTATGCTCAGGCTACAGA-3′	5′-GCATTGGCGATTTCATTGGTC-3′

### Statistical analysis

The relative mRNA expression data were analyzed with one-way ANOVA followed by Fisher’s least significant difference (LSD) comparison. The mRNA data were performed with GraphPad Prism software version 6.0 (GraphPad Software, Inc., La Jolla, CA). *P*-value < 0.05 indicates there’s difference and *P*-value < 0.01 indicates significant difference. The statistical method of bioinformatics analysis was completely performed by R software and specifically described in the part of each method.

## Results

### Basic datasets of OA and RA

**Fig. 1:**
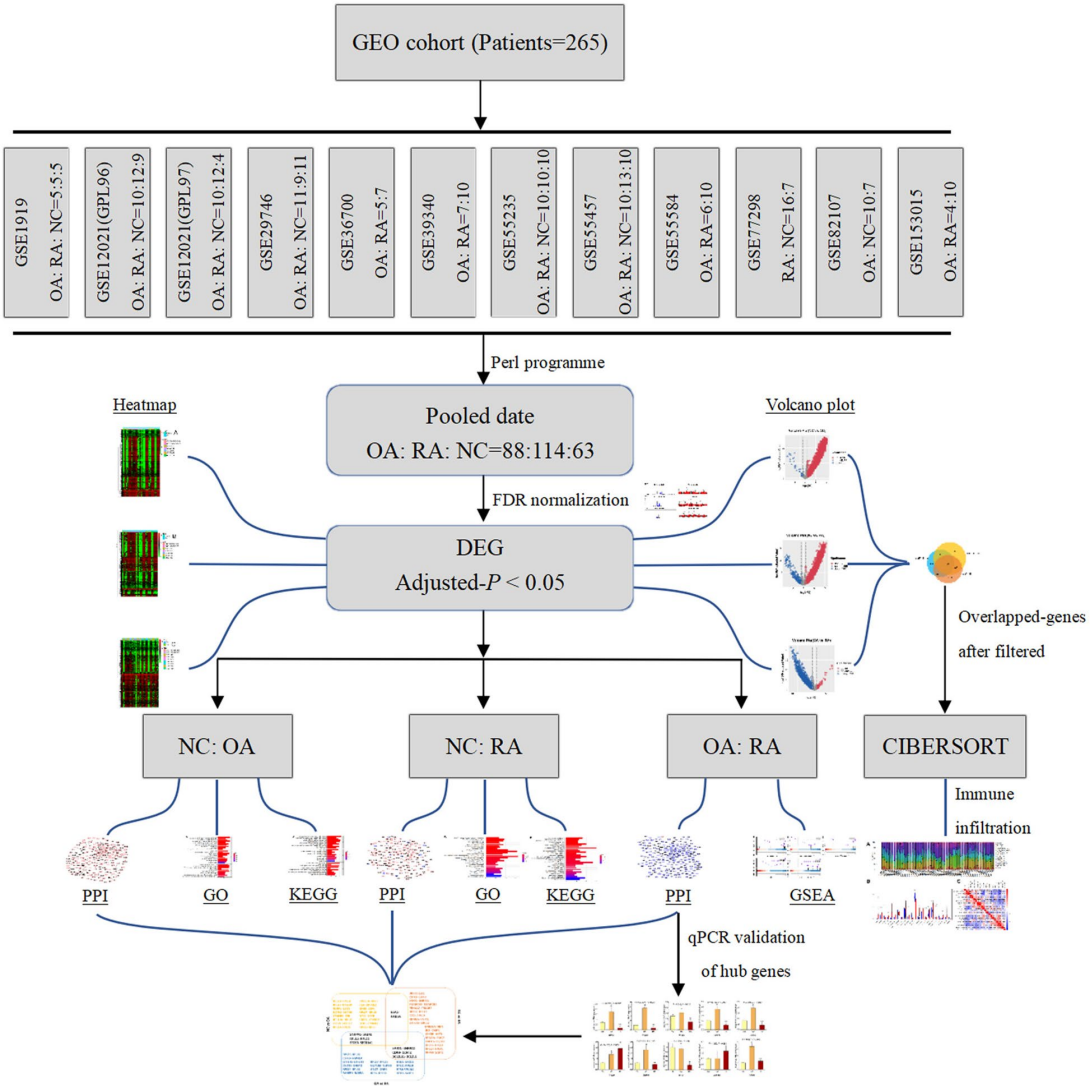
Schematic diagram of the whole study

**Fig. 2:**
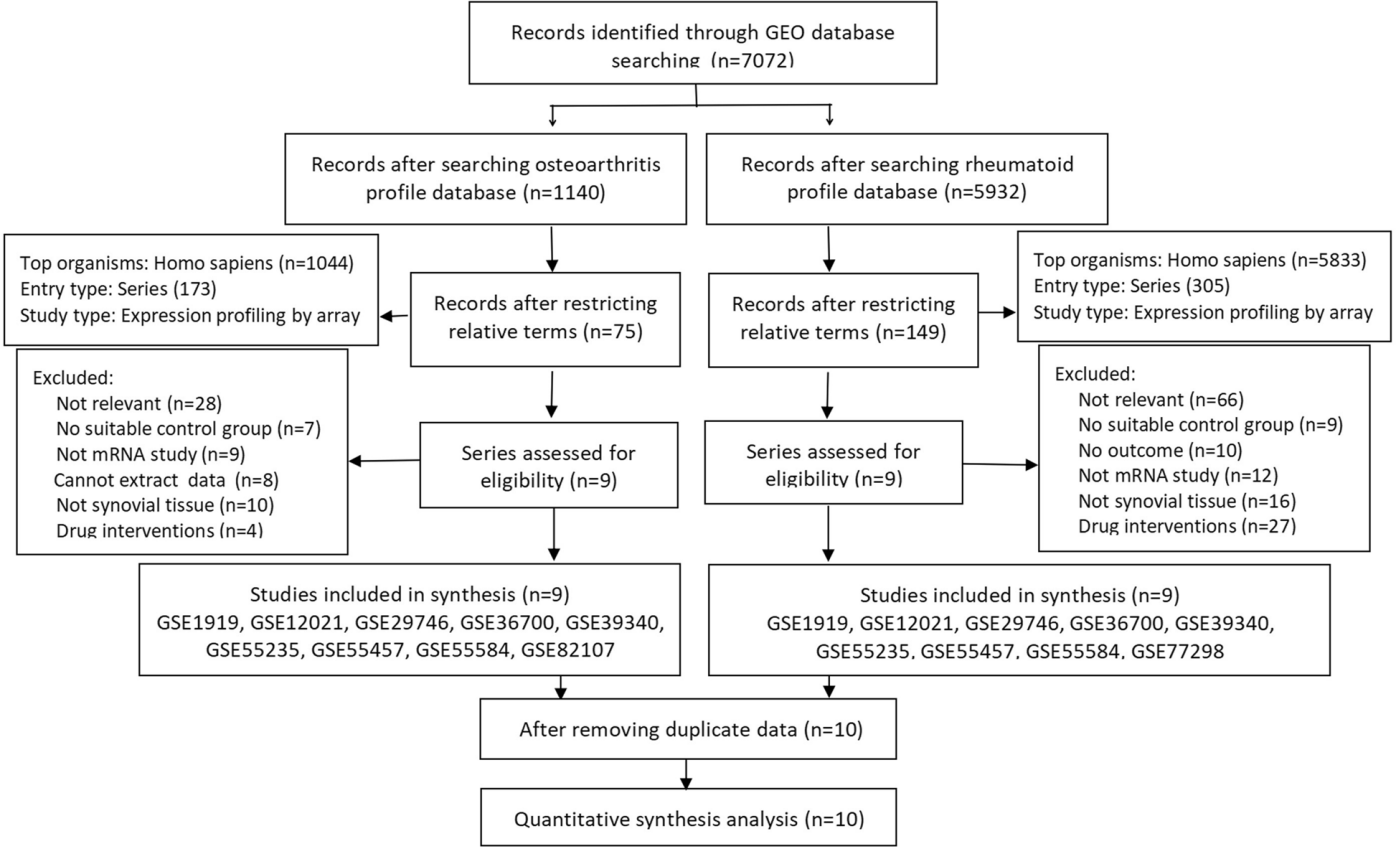
Flow chart of GEO series accession screening

In this study, we performed an integrative analysis of gene expression and the entire workflow was shown in Fig. 1. A total of 11 expression data sets of OA, RA, and NC were downloaded. Their GEO accession numbers are GSE1919, GSE12021 (GPL96 and GPL97), GSE29746, GSE36700, GSE39340, GSE55235, GSE55457, GSE55584, GSE77298, and GSE82107. To conclude, 265 human synovial membrane samples were enrolled in this study with all data merged. Among them, there were 88 OA samples, 114 RA samples and 63 negative control (NC) samples. Figure 2 depicted the relevant details of the selection process. All relevant information of these eleven GEO datasets was showed in Table 3.

**Table 3:** Summary information of studies included in analysis

GEO accession	Author	Public date	Platform	OA: RA: NC
GSE1919	Ungethuem U	Nov 04, 2004	GPL91 [HG_U95A] Affymetrix Human Genome U95A Array	5:5:5
GSE12021 (GPL96)	Huber R	Sep 02, 2008	GPL96 [HG-U133A] Affymetrix Human Genome U133A Array	10:12:9
GSE12021 (GPL97)	Huber R	Sep 02, 2008	GPL97 [HG-U133B] Affymetrix Human Genome U133B Array	10:12:4
GSE29746	Del Rey MJ	Oct 25, 2011	GPL4133 Agilent-014850 Whole Human Genome Microarray 4 × 44 K G4112F (Feature Number version)	11:9:11
GSE36700	Lauwerys BR	Mar 27, 2012	GPL570 [HG-U133_Plus_2] Affymetrix Human Genome U133 Plus 2.0 Array	5:7:0
GSE39340	Chang X	Oct 22, 2012	GPL10558 Illumina HumanHT-12 V4.0 expression beadchip	7:10:0
GSE55235	Woetzel D	Feb 21, 2014	GPL96 [HG-U133A] Affymetrix Human Genome U133A Array	10:10:10
GSE55457	Woetzel D	Mar 05, 2014	GPL96 [HG-U133A] Affymetrix Human Genome U134A Array	10:13:10
GSE55584	Woetzel D	Mar 05, 2014	GPL96 [HG-U133A] Affymetrix Human Genome U135A Array	6:10:0
GSE77298	Broeren MG	Jan 27, 2016	GPL570 [HG-U133_Plus_2] Affymetrix Human Genome U133 Plus 2.0 Array	0:16:7
GSE82107	de Vries M	Jun 02, 2016	GPL570 [HG-U133_Plus_2] Affymetrix Human Genome U133 Plus 2.0 Array	10:0:7
GSE153015	Triaille C	Jun 22, 2020	GPL570[HG-U133_Plus_2] Affymetrix Human Genome U133 Plus 2.0 Array	4:10:0

*GEO* Gene Expression Omnibus, *OA* osteoarthritis, *RA* rheumatoid arthritis, *NC* normal control

### DEGs screening

**Fig. 3:**
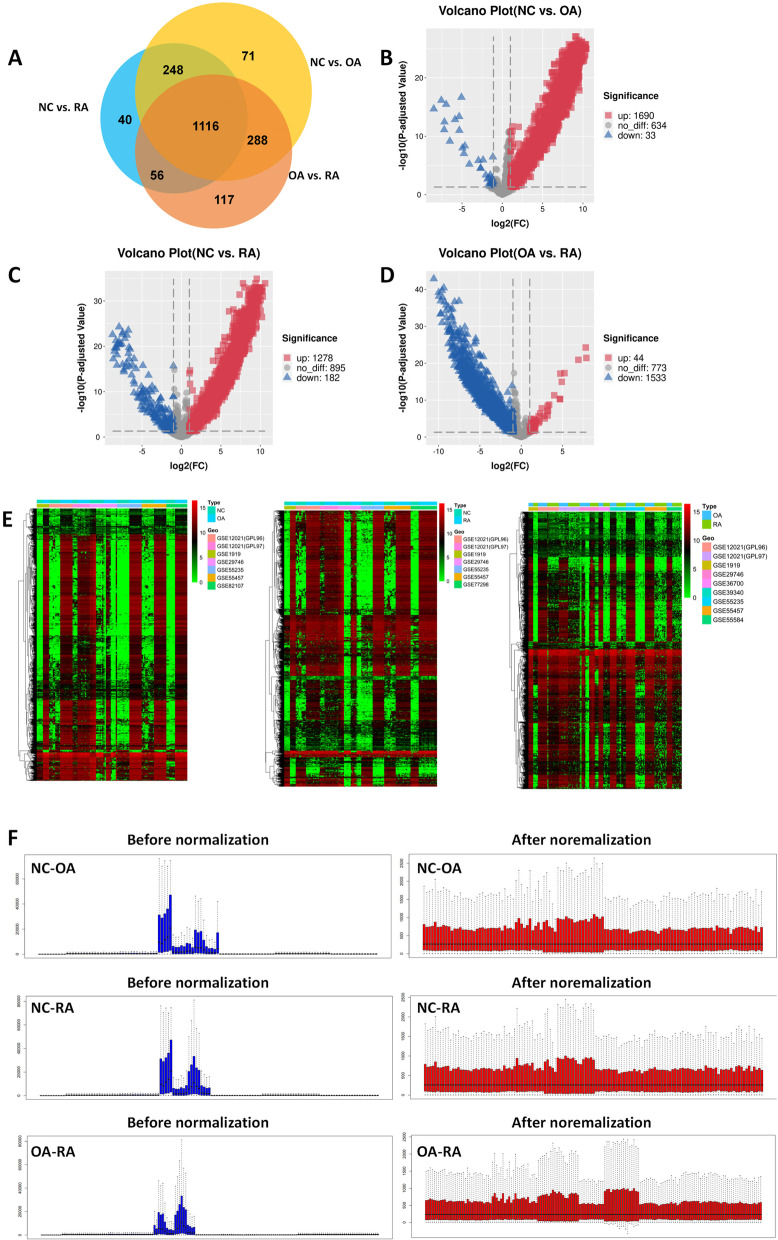
The process of DEGs screening. **A** Venn diagram of shared DEGs between OA, RA and NC. The yellow circle represents DEGs in NC and OA, the blue circle represents DEGs in health and RA, and the red circle represents DEGs in OA and RA. **B** Volcano plot of all DEGs between health and OA. **C** Volcano plot of all DEGs between health and RA. **D** Volcano plot of all DEGs between OA and RA. **E** Heatmap of DEGs. The row represents the expression of DEG, and the column represents the samples. The different color scale represents the different expression levels. **F** Before and after normalization of DEGs in three groups

As shown in Fig. 3A, a total of 1723 and 1460 DEGs were identified in OA and RA samples, respectively, compared with NC samples. Within the DEGs data, a total of 1690 up-regulated genes and 33 down-regulated genes were indentified in OA group compared with NC group (Fig. 3B). While comparing RA group with NC group, 1278 up-regulated genes and 182 down-regulated genes were indentified in the DEGs data (Fig. 3C). As shown in Fig. 3D, a total of 1116 genes were indentified in DEGs intersection from OA group and RA group, while all compared with NC group independently. Nevertheless, comparing OA with RA group, 1577 DEGs (OA vs. RA: 44 up-regulated and 1533 down-regulated) were identified. Three heatmap diagrams of DEGs were represented in Fig. 3E and visually showing that these similar DEGs could significantly distinguish one from the other. The top 5 up- and down-regulated DEGs of the three groups were shown in Table 4. The probe expression matrix files downloaded from the GEO database were normalized and the three sets of data were shown in Fig. 3F.

**Table 4:** Top 5 up- and downregulated DEGs between OA, RA and NC

Term	NC vs. OA				NC vs. RA				OA vs. RA			
	ID	Symbol	logFC	Adj.*P*.Val	ID	Symbol	logFC	Adj.*P*.Val	ID	Symbol	logFC	Adj.*P*.Val
Up-regulated	4613	MYCN	9.180080878	8.02E-28	8935	SKAP2	9.553082085	1.26E-35	23,094	SIPA1L3	7.79814072	5.46E-25
	6598	SMARCB1	9.03478793	8.02E-28	10,745	PHTF1	9.39043259	1.29E-34	1404	HAPLN1	7.87947808	3.69E-22
	23,365	ARHGEF12	9.51514144	3.28E-27	22,925	PLA2R1	7.801574317	1.29E-34	5074	PAWR	6.908165575	1.22E-21
	5137	PDE1C	9.117030454	4.96E-27	9488	PIGB	9.97554221	1.04E-33	10,100	TSPAN2	5.2303578	4.83E-18
	8697	CDC23	9.841678124	6.56E-27	9294	S1PR2	9.470029664	1.04E-33	22,999	RIMS1	4.725490721	6.50E-18
Down-regulated	5340	PLG	-5.068734955	2.10E-17	5208	PFKFB2	-7.906263354	5.11E-25	954	ENTPD2	-10.57701223	1.29E-43
	7368	UGT8	-7.56536003	7.07E-17	9024	BRSK2	-6.728489168	2.71E-24	4846	NOS3	-9.366652871	3.92E-41
	3575	IL7R	-6.966596378	3.51E-16	23,308	ICOSLG	-7.439436875	8.41E-24	6875	TAF4B	-9.588028779	2.08E-40
	1404	HAPLN1	-8.500030865	2.05E-15	2676	GFRA3	-7.481477156	2.29E-23	2395	FXN	-9.989764717	6.81E-40
	22,999	RIMS1	-5.348208851	4.24E-14	6875	TAF4B	-8.729849528	2.50E-23	777	CACNA1E	-9.95687779	7.39E-39

*DEGs* differentially expressed genes, *OA* osteoarthritis, *RA* rheumatoid arthritis, *NC* normal control, *logFC* log fold change

### GO pathway of OA-specific and RA-specific DEGs

**Fig. 4:**
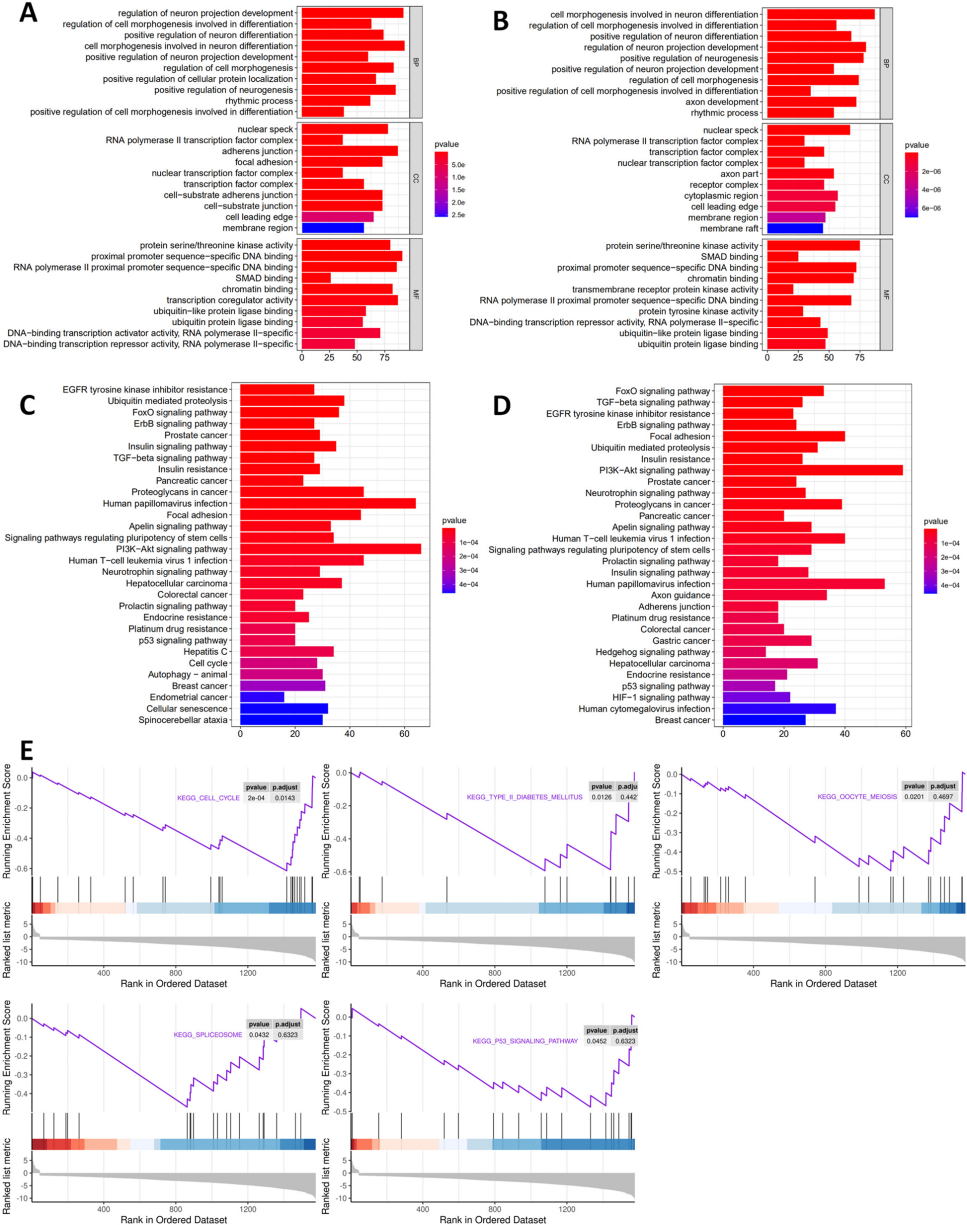
GO enrichment, KEGG terms and GSEA pathway analysis of DEGs. **A** GO terms in the OA-related enrichment analysis of DEGs. **B** GO terms in the RA-related enrichment analysis of DEGs. **C** OA-related pathway enrichment analysis of DEGs. **D** RA-related pathway enrichment analysis of DEGs. **E** GSEA analysis of DEGs between OA and RA

GO annotations analysis showed that OA-specific DEGs (Table 5, Fig. 4A) were predominantly enriched in regulation of neuron projection development, regulation of cell morphogenesis involved in differentiation, and positive regulation of neuron differentiation (for BP); nuclear speck, RNA polymerase II transcription factor complex, and adherens junction (for CC); protein serine/threonine kinase activity, proximal promoter sequence-specific DNA binding, and RNA polymerase II proximal promoter sequence-specific DNA binding (for MF). RA-specific DEGs (Table 5, Fig. 4B) were primarily enriched in cell morphogenesis involved in neuron differentiation, regulation of cell morphogenesis involved in differentiation, and positive regulation of neuron differentiation (for BP); nuclear speck, RNA polymerase II transcription factor complex, and transcription factor complex (for CC); protein serine/threonine kinase activity, SMAD binding, and proximal promoter sequence-specific DNA binding (for MF).

**Table 5:** GO pathway analysis of differentially expressed genes

Term		ID	Description	Adj.*P*.Val	Count
OA-specific	BP	GO:0,010,975	regulation of neuron projection development	3.97E-10	92
		GO:0,010,769	regulation of cell morphogenesis involved in differentiation	3.97E-10	63
		GO:0,045,666	positive regulation of neuron differentiation	6.79E-09	74
	CC	GO:0,016,607	nuclear speck	4.51E-10	78
		GO:0,090,575	RNA polymerase II transcription factor complex	2.64E-09	37
		GO:0,005,912	adherens junction	2.12E-08	87
	MF	GO:0,004,674	protein serine/threonine kinase activity	6.24E-10	80
		GO:0,000,987	proximal promoter sequence-specific DNA binding	6.24E-10	91
		GO:0,000,978	RNA polymerase II proximal promoter sequence-specific DNA binding	3.27E-09	86
RA-specific	BP	GO:0,048,667	cell morphogenesis involved in neuron differentiation	8.82E-10	87
		GO:0,010,769	regulation of cell morphogenesis involved in differentiation	1.52E-09	56
		GO:0,045,666	positive regulation of neuron differentiation	1.52E-09	68
	CC	GO:0,016,607	nuclear speck	1.53E-08	67
		GO:0,090,575	RNA polymerase II transcription factor complex	1.17E-06	30
		GO:0,005,667	transcription factor complex	1.15E-05	46
	MF	GO:0,004,674	protein serine/threonine kinase activity	2.69E-11	75
		GO:0,046,332	SMAD binding	4.46E-08	25
		GO:0,000,987	proximal promoter sequence-specific DNA binding	2.44E-06	72

*GO* Gene Ontology, *OA* osteoarthritis, *RA* rheumatoid arthritis

### KEGG pathway of OA-specific and RA-specific DEGs

As shown in Fig. 4C, D, and Table 6, the top three significantly enriched signaling pathways of OA-specific DEGs were EGFR tyrosine kinase inhibitor resistance, ubiquitin mediated proteolysis, and FoxO signaling pathway. KEGG pathway analysis of DEGs showed that FoxO signaling pathway, TGF-beta signaling pathway, and EGFR tyrosine kinase inhibitor resistance were the top three significantly enriched pathways in RA.

**Table 6:** KEGG analysis of differentially expressed genes

Term	ID	Description	Adj.*P*.Val	Count
OA-specific	hsa01521	EGFR tyrosine kinase inhibitor resistance	7.94E-06	27
	hsa04120	Ubiquitin mediated proteolysis	7.94E-06	38
	hsa04068	FoxO signaling pathway	1.88E-05	36
	hsa04012	ErbB signaling pathway	2.08E-05	27
	hsa05215	Prostate cancer	2.59E-05	29
	hsa04910	Insulin signaling pathway	9.18E-05	35
	hsa04350	TGF-beta signaling pathway	1.10E-04	27
	hsa04931	Insulin resistance	1.53E-04	29
	hsa05205	Proteoglycans in cancer	1.53E-04	45
RA-specific	hsa04068	FoxO signaling pathway	2.39E-05	33
	hsa04350	TGF-beta signaling pathway	3.84E-05	26
	hsa01521	EGFR tyrosine kinase inhibitor resistance	3.84E-05	23
	hsa04012	ErbB signaling pathway	3.84E-05	24
	hsa04120	Ubiquitin mediated proteolysis	1.12E-04	31
	hsa04510	Focal adhesion	1.12E-04	40
	hsa04931	Insulin resistance	2.05E-04	26
	hsa04151	PI3K-Akt signaling pathway	2.23E-04	59
	hsa05215	Prostate cancer	2.23E-04	24

*KEGG* Kyoto Encyclopedia of Genes and Genomes, *OA* osteoarthritis, *RA* rheumatoid arthritis

### GSEA of involved signaling pathways

GSEA analysis unveiled that DEGs between OA and RA were significantly enriched in several pathways (Fig. 4E). We found that RA was significantly associated with the high expression of cell cycle (*P* = 0.0143), type II diabetes mellitus (*P* = 0.0126), oocyte meiosis (*P* = 0.0201), spliceosome (*P* = 0.0432), and p53 signaling pathway (*P* = 0.0452). OA, in contrast, had lower activities in these pathways.

### PPI network and modules

**Fig. 5:**
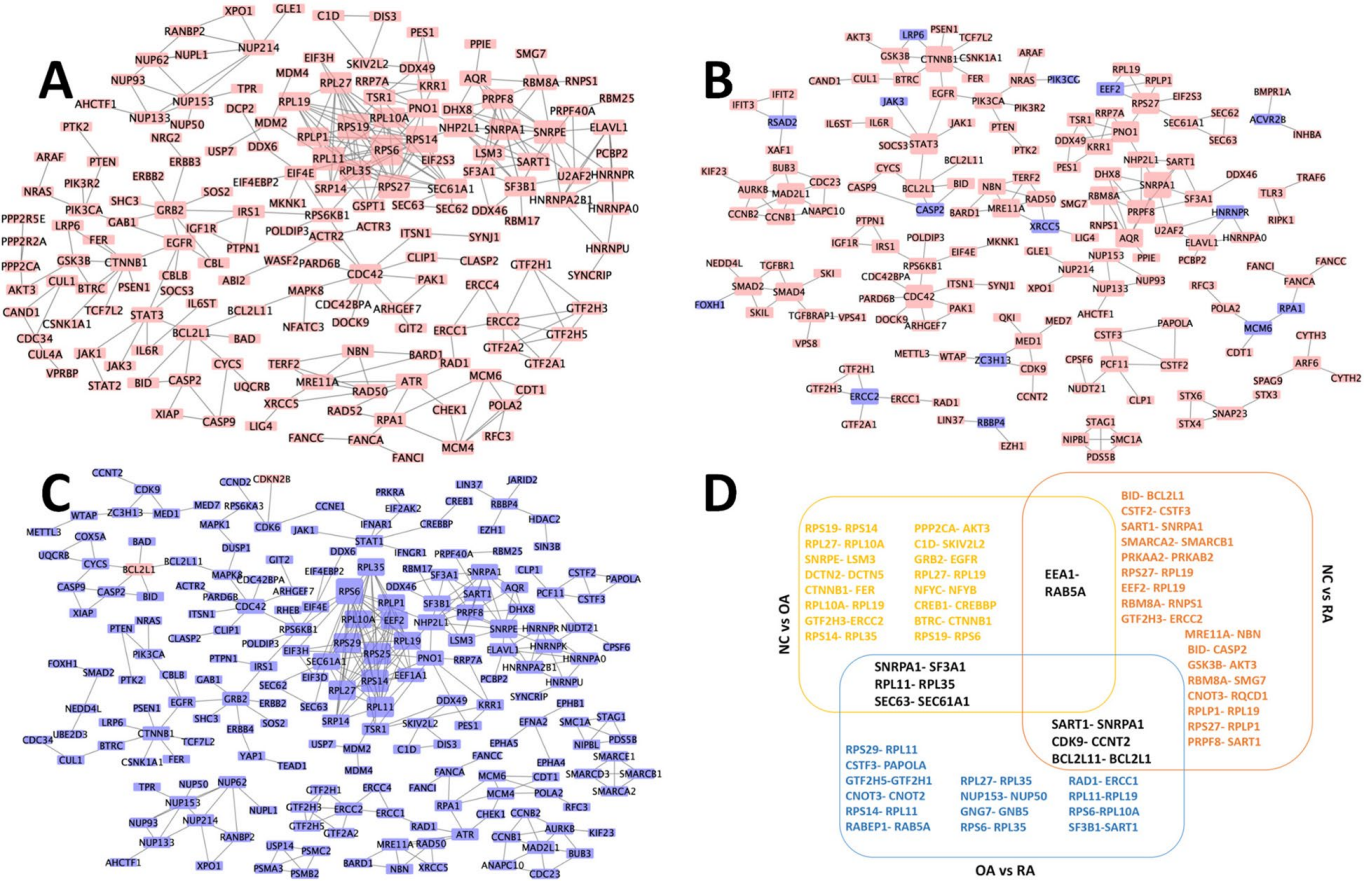
Hub genes of OA-specific and RA-specific PPI networks. **A** OA relative PPI network. **B** RA relative PPI network. **C** PPI network constructed by DEGs between OA and RA. **D** Three groups of top 20 connections in PPI network. The red and blue rectangles represent the proteins encoded by up or down-regulated DEGs, respectively. The larger rectangles indicate the higher degree in networks

The OA-specific PPI network consisted of 391 nodes and 483 edges (Fig. 5A), the top five hub nodes of which were RPS6 (ribosomal protein S6, degree = 19), RPS14 (ribosomal protein S14, degree = 16), RPS19 (ribosomal protein S19, degree = 15), RPL11 (ribosomal protein L11, degree = 13) and RPS27 (ribosomal protein S27, degree = 13). The RA-specific PPI network consisted of 273 nodes and 259 edges (Fig. 5B), the top five hub nodes of which were CTNNB1 (catenin beta 1, degree = 8), SNRPA1 (small nuclear ribonucleoprotein polypeptide A 1, degree = 8), CDC42 (cell division cycle 42, degree = 7), PRPF8 (pre-mRNA processing factor 8, degree = 7) and MAD2L1 (mitotic arrest deficient 2 like 1, degree = 6). The PPI network constructed by DEGs between OA and RA was shown in Fig. 5C. In the top 20 connections of these three PPI networks, overlapped genes were shown in Fig. 5D. The top 10 hub genes identified between OA and RA (*RPS6*, *RPS14*, *RPS25*, *RPL11*, *RPL27*, *RPS29*, *SNRPE*, *EEF2*, *RPL10A* and *RPL19*) were selected for further experimental verification.

### Immune infiltration

**Fig. 6:**
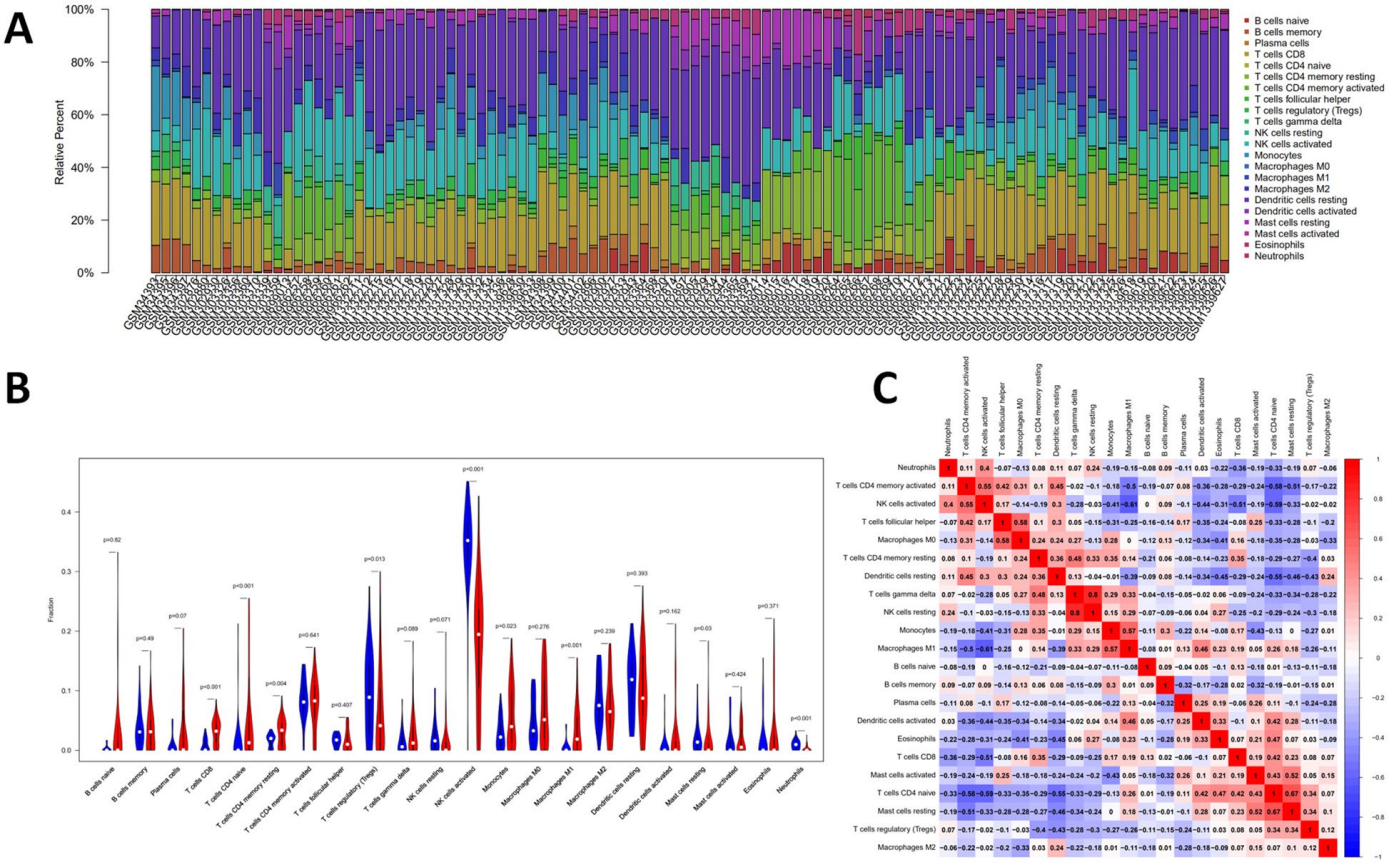
The profiles of immune infiltration between OA and RA. **A** The percentage of 22 subpopulations of IICs. **B** Violin plot showing the difference of immune infiltration between OA (Marked as blue color on the left) and RA (Marked as red color on the right). Adjusted-*P* values < 0.05 were considered as statistical significance. **C** The correlation analysis of the 22 immune cells. The red color represents the positive relationship between two immune cells and the blue color represents the negative relationship between two immune cells. The darker color indicates the stronger correlation

In this study, the IICs in 22 subpopulations of immune cells were evaluated. As shown in Fig. 6A, the proportions and percentage of immune cells were depicted. The heatmap showed that the levels of NK cells activated, dendritic cells resting, T cells regulatory (Tregs) and macrophages M2 were relatively high in the 22 immune cells. The violin plot (Fig. 6B) indicated that the proportions of B cells memory (*P* = 0.005), T cells regulatory (Tregs) (*P* = 0.003) and NK cells activated (*P* < 0.001) were relatively high in OA synovial tissues compared with that in RA synovial tissues, while B cells naive (*P* < 0.001), plasma cells (*P* = 0.004), T cells CD4 naive (*P* = 0.018), T cells CD4 memory activated (*P* = 0.001), dendritic cells activated (*P* = 0.003) and eosinophils (*P* < 0.001) were relatively low in OA. The quantified contrast of the distribution of IICs subsets between OA and RA synovial tissues was shown in Fig. 6C. Interestingly, the result showed that there was a positive correlation between monocytes and B cells memory, and a negative correlation between dendritic cells activated and T cells CD8.

### Validation of the key genes

**Fig. 7:**
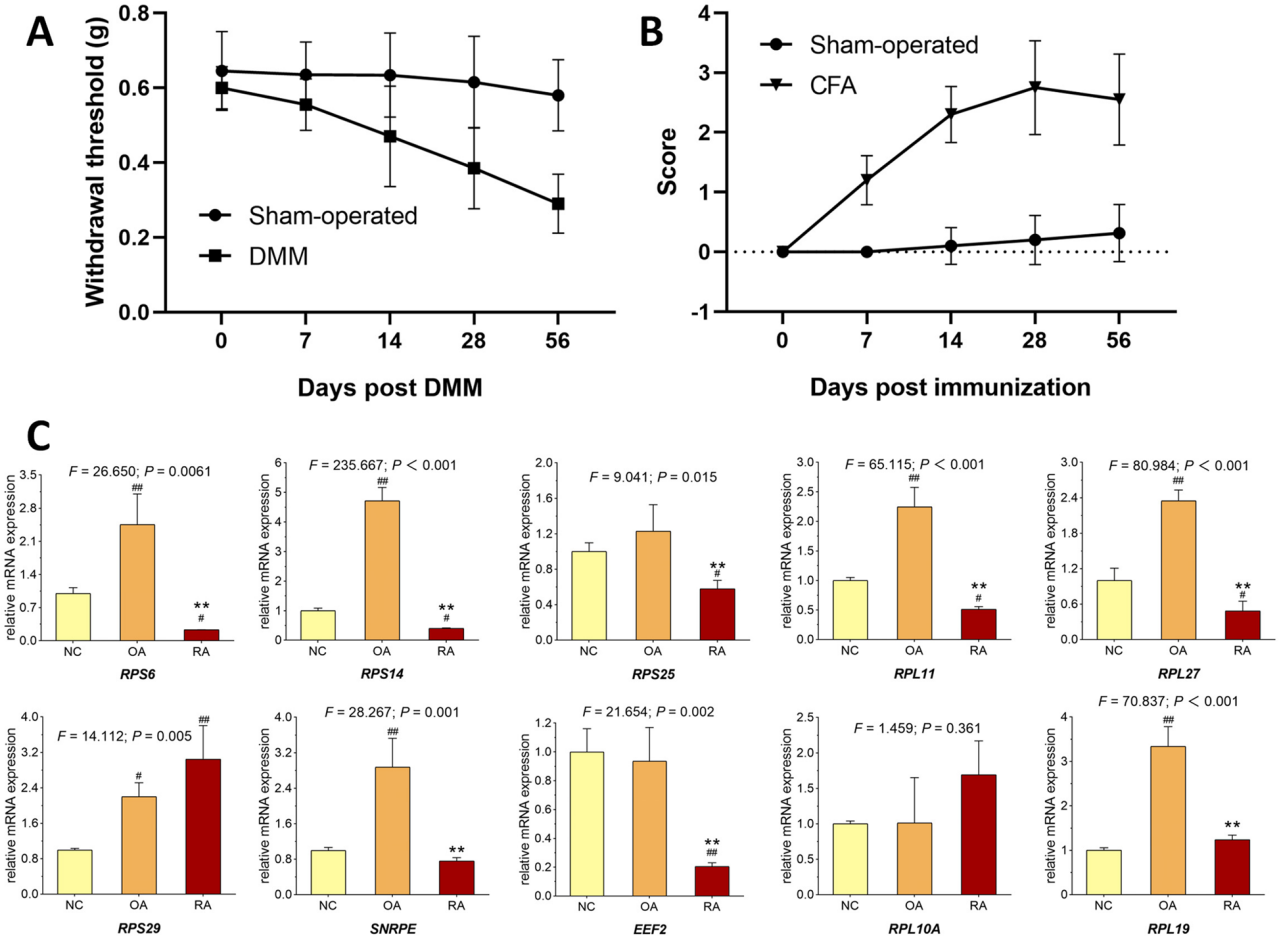
Animal experiments. **A** Assessing the OA model using the mechanical allodynia. **B** Arthritis index score to assess RA model. **C** Relative mRNA expressions of target genes in synovial membrane from NC, OA and RA mice. Values are presented as mean ± SD. ^#^*P* < 0.05 or ^##^*P* < 0.01 vs. NC group; **P < 0.01 vs. OA group

After OA and RA mouse models were successfully established (Fig. 7A and B), synovial tissue from OA, RA and NC mice were collected. qPCR assays were performed in these tissues to verify the expression of the identified top 10 hub genes. Based on the qPCR results, the expression of *RPS6*, *RPS14*, *RPS25*, *RPL11*, *RPL27*, *SNRPE*, *EEF2* and *RPL19* (Fig. 7C) were significantly down-regulated in RA mice compared with that in OA mice (*P* < 0.05). All validations except *RPS29* and *RPL10A* were consistent with the microarray data and analytical results in this study.

## Discussion

Recently, with increasing aging populations and chronic conditions, both OA and RA have become the most common causes of musculoskeletal-related chronic joint disorders in elders [27, 28]. Although multiple diagnostic and therapeutic approaches are available for OA and RA, the clinical outcomes are still not satisfactory [29]. Genomic studies have been widely used to enhance the diagnosis and treatment of many diseases, including OA and RA [14]. To investigate the mechanisms underlying OA and RA, we systematically analyzed these multiple microarray data sets using an integrated method with the largest sample size so far.

A total of 1723 OA-specific DEGs (1690 up- and 33 down-regulated genes) were screened. Among them, 1683 overlapping genes were obtained, and 71 DEGs were acquired, such as *TPM2*, *NCAM2*, and *MFHAS1*, after eliminating RA-related genes. Ubiquitin was significantly enriched in OA-related GO terms and pathways of DEGs. The study found 1460 RA-specific DEGs in total (1278 up- and 182 down-regulated genes). A total of 40 RA-specific genes were acquired, such as *CUX1*, *KANK1,* and *MBTPS2*. These DEGs may be important to explain the occurrence and progression of OA and RA. Moreover, three PPI networks of multigroup DEGs were established using STRING and overlapped connected nodes were constructed to further explore the deep relationship of OA and RA. The top 10 distinguished hub genes, including *RPS6*, *RPS14*, *RPS25*, *RPL11*, *RPL27*, *SNRPE*, *EEF2*, *RPS29, RPL10A* and *RPL19*, were identified and verified in OA and RA synovial samples. The qPCR results showed that, except *RPS29* and *RPL10A*, the other 8 hub genes were consistent with our bioinformatic analysis. The inconsistent expression pattern of *RPS29* and *RPL10A* might due to different species (bioinformatic data in human and validation in mice), which finally lead to minor differences in certain genes.

Phosphorylating ribosomal S6 protein kinase (*RPS6*) was the core gene in PPI network and we discovered inextricably linked with pathways in this study. *RPS6* participated in the mammalian target of rapamycin (mTOR) signal pathway and was triggered by anabolic signals [30]. mTOR is well known upstream regulator of autophagy. Autophagy has been demonstrated to be responsible for cellular homeostasis and metabolic regulation, and plays an essential role in the development of structural changes and aging in cartilage [31, 32]. Evidences suggested that the expression of mTOR increased in OA joint cartilage, and genetic or pharmaceutical inhibition of mTOR signaling could activate autophagy and protect mice against OA [33, 34]. KEGG pathway analysis identified top enriched PI3K/Akt signaling pathway had also been reported to involve in OA pathology via modulating autophagy [35, 36].

Ubiquitin mediated proteolysis is one of top three pathways enriched in OA-specific DEGs. Previous studies suggested that the biological process of ubiquitination and the proteasome led to OA by regulating the expression of inflammatory cytokines [37]. Frank et al. found that ubiquitin was an integral part of ubiquitin-like proteins, which covered the Small Ubiquitin-related Modifiers (SUMO) [38]. Histological analysis of synovial tissue obtained from OA and RA patients demonstrated a correlation between SUMO and MMP13, a well-established catabolic factor for cartilage degeneration, and SUMO-2/3 under TNF-α stimulation could selectively affect MMP expression via the NF-κB pathway [39].

EGFR signaling pathway, enriched as EGFR tyrosine kinase inhibitor resistance in both OA- and RA-specific DEGs, was necessary for regulating the proliferation, survival and biological properties of joint superficial chondrocytes [40]. It also played a pivotal role in growth plate development and secondary ossification center formation [41, 42]. Sun et al. identified a subgroup of OA patients displaying high expression levels of EGFR, and suggested that EGFR could be a promising target for OA therapy [43]. Another study also suggested that modulation of the EGFR pathway promoted mesochondrium synthesis and suppressed OA cartilage degradation [44].

FoxO signaling pathway was also enriched in both OA- and RA-specific DEGs. Lee et al. identified FoxO3 activity as a severity marker for RA, via modulating cytokine production in monocytes, and a FoxO3a haplotype was related to erosion scores in adult RA [45]. Some studies reported that in RA joint synovial tissue, modulation of known transcriptional FoxO-related factors played a role in integrating inflammatory stimuli to regulate cell survival and apoptosis [46]. Besides, FoxO signaling also plays a vital role in OA. In humans and mice, dysregulated FoxO expression and activation had been reported to be involved in cartilage aging and OA, and FoxO deletion in mice led to more severe cartilage damage [47, 48].

TGF-beta signaling pathway was enriched only in RA-specific DEGs based on this study. As known that TGF-beta executed different actions in RA and OA synovial fibroblasts. Tetsuji Kobata found that TGF-beta reduced OPG production by RA synovial fibroblasts, but dose-dependently increased OPG secretion in OA synovial fibroblasts [49]. Another study reported that compared with fibroblast-like synoviocytes (FLS) derived from OA patients, RA FLS displayed TGF-beta-dependent overexpression of non-receptor protein tyrosine phosphatase 14 (PTPN14), which promoted TGF-beta canonical signaling in turn [50]. Xu Cao et. al had reported that in RA mouse and rat model, aberrant activation of TGF-beta in the subchondral bone was involved at the onset of RA joint cartilage degeneration, and they insisted that the pathogenesis of cartilage degeneration in RA and OA may converge on the aberrant activation of TGF-beta in the subchondral bone [51]. Hence, TGF-beta signaling might contribute to distinguished diagnosis and treatment between OA and RA.

Previous studies and experiments validation had shown that top genes and pathways enriched in our analysis were quite reliable. Nevertheless, our study still had some limitations that needed to be improved. First, the GEO database in this study was the only resource to acquire related data and perform data mining. Second, both OA and RA mouse models could not entirely duplicate the occurrence of OA and RA in humans, so the qPCR validation might to some extent showed inconsistency with human GEO data. Thus, a multicenter, larger-scale clinical survey was an indispensable step for further research.

## Conclusions

In this study, multiple similar genes and signaling pathways were revealed to be simultaneously involved in both OA and RA. Then, genes and pathways present in different patterns offered us new clues to discover potential biomarkers and underlying molecular mechanisms for OA and RA, respectively. Our findings might also contribute to the differential diagnosis of OA and RA focusing on synovial membrane, though large samples and sophisticated algorithms are necessitated for further research.

## Data Availability

The datasets used and/or analyzed during the current study are available from GEO database (https://www.ncbi.nlm.nih.gov/geo/) and the corresponding author on reasonable request.
